# Mechanistic and therapeutic insights into the function of different cell death modalities in rheumatoid arthritis: emphasis on the crosstalk with non-coding RNAs

**DOI:** 10.3389/fimmu.2025.1620209

**Published:** 2025-07-23

**Authors:** Jianting Wen, Jian Liu, Lei Wan, Fanfan Wang

**Affiliations:** ^1^ Department of Rheumatology and Immunology, First Affiliated Hospital of Anhui University of Chinese Medicine, Hefei, Anhui, China; ^2^ Institute of Rheumatology, Anhui Academy of Chinese Medicine, Hefei, Anhui, China; ^3^ Anhui Province Key Laboratory of Modern Chinese Medicine, Department of Internal Medicine Application Foundation Research and Development, Hefei, Anhui, China

**Keywords:** rheumatoid arthritis, different cell death modalities, non-coding RNAs, mechanistic and therapeutic, TCM

## Abstract

Rheumatoid arthritis (RA), a prevalent autoimmune disorder, imposes a substantial burden on global health due to its progressive disability and compromised patient well-being. Although the precise etiology of this condition is still not fully understood, current research implicates intricate interactions between dysregulated immune cells and pro-inflammatory mediators. Recent scientific advancements have highlighted the pathogenic significance of programmed cell death (PCD) mechanisms (including spanning apoptosis, autophagy, ferroptosis, necroptosis, senescence, and pyroptosis) in RA pathophysiology. Emerging evidence has established these cellular demise pathways as critical contributors to synovial inflammation and joint destruction. This comprehensive analysis systematically examined the mechanistic involvement of distinct cell death modalities in RA development, with particular focus on their regulatory interplay with non-coding RNAs (ncRNAs). Furthermore, the emerging therapeutic potential of traditional Chinese medicine (TCM) formulations in modulating these cell death networks was evaluated, ultimately proposing novel translational frameworks for targeted RA intervention.

## Introduction

Rheumatoid arthritis (RA) is a long-lasting autoimmune disorder that primarily presents with erosive and symmetrical polyarthritis, which can not only cause deformities in the joints and loss of function but may also affect several bodily systems, including the heart, lungs, and nervous system (1, 2). The global occurrence rate of RA ranges between 0.5% and 1%, with women experiencing RA 2–3 times more frequently than men, and this frequency is on the rise in women (3). Although the exact causes and progression of RA are unknown, it is widely believed by researchers that a combination of factors (including genetic, immune, hormonal, environmental, smoking, and infectious elements) contribute to RA development (4). Recent research has highlighted that non-coding RNAs (ncRNAs), including microRNAs (miRNAs), long non-coding RNAs (lncRNAs), and circular RNAs (circRNAs), play central roles as epigenetic regulators in the pathogenesis of RA (5). These ncRNAs orchestrate complex gene regulatory networks that govern immune cell activation, inflammatory mediator production, and, notably, the activation of various programmed cell death (PCD) pathways (such as pyroptosis, necroptosis, apoptosis, and ferroptosis) (6). The dynamic crosstalk between ncRNAs and cell death mechanisms represents a novel frontier in understanding RA pathophysiology and developing precision therapeutics.

RA treatment focuses on clinical remission, decreasing inflammation and joint destruction, and boosting the patient’s quality of life (7). At present, the principal medications used to treat RA include nonsteroidal anti-inflammatory drugs (NSAIDs), disease-modifying antirheumatic drugs (DMARDs), biological agents, and corticosteroids (8). However, extended use of Western pharmaceuticals has shortcomings, including harmful gastrointestinal reactions, damage to the liver and kidneys, condition worsening after discontinuation, and high costs (9). Consequently, understanding the pathogenesis of RA and pinpointing possible therapeutic targets is essential.

**Figure 1:**
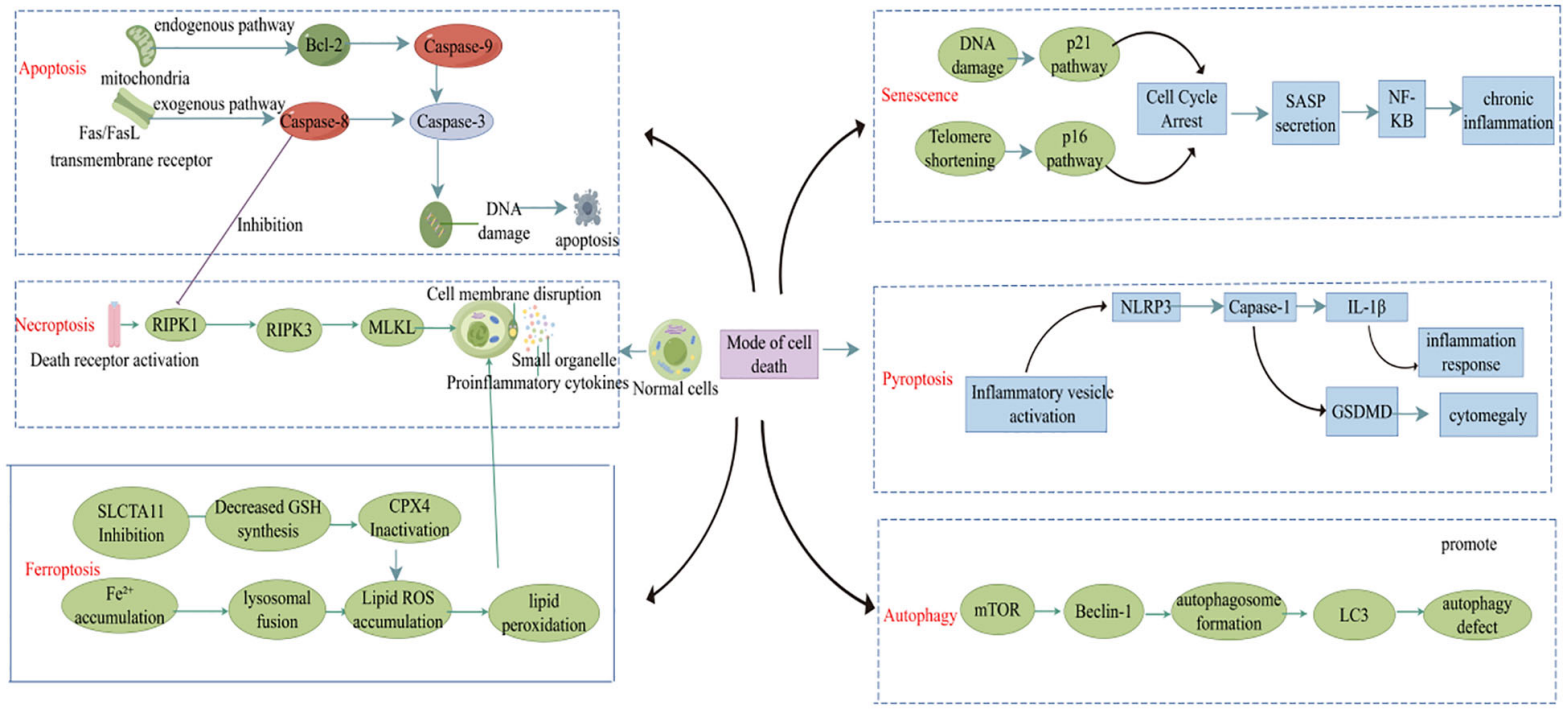
Different cell death modalities in RA.

Cell death plays a crucial role in maintaining tissue balance, but improper regulation can trigger or worsen diseases (such as RA) (10). In recent years, with the deepening of research on cell death, various PCD mechanisms [such as apoptosis, pyroptosis, autophagy, and ferroptosis (**Figure 1**)] have been established as contributors to RA pathogenesis by controlling the dynamic balance within the immune microenvironment (11). Dysfunctional apoptotic pathways may lead to the release of autoantigens and the breakdown of immune tolerance, whereas necroptosis exacerbates local inflammation by releasing damage-associated molecular patterns (DAMPs) that activate fibroblast-like synoviocytes (FLS) and macrophages. Pyroptosis is mediated by gasdermin protein-induced membrane permeabilization and IL-1β secretion, which is closely linked to the inflammatory cascade within the RA joints. These senescent cells release pro-inflammatory cytokines that drive synovial hyperplasia, pannus formation, and joint destruction. Furthermore, the role of autophagy-related genes in balancing Th17/Treg cell differentiation and ferroptosis-driven oxidative stress to promote subchondral bone erosion highlights the intricate interplay between cell death mechanisms and RA pathology. Elucidating the spatiotemporal regulation of these death pathways in RA might reveal novel therapeutic targets and inform precise intervention strategies.

The pathogenesis of RA involves multidimensional dysregulation of gene expression, with aberrant regulation of ncRNAs, especially circRNAs and lncRNAs, which are pivotal molecular hubs for deciphering RA-related immune dysregulation and joint destruction (5, 12). By leveraging their covalently closed circular structure and high stability, circRNAs dynamically regulate inflammatory processes in RA by acting as competing endogenous RNAs (ceRNAs) to sequester miRNAs or directly bind RNA-binding proteins (13). In contrast, lncRNAs broadly participate in RA pathophysiology through chromatin remodeling, transcriptional regulation, and epigenetic modifications (14). The crosstalk between ncRNAs and different cell death pathways plays a key role in the pathogenesis of RA, forming the core driving factor of the “inflammation-death-repair imbalance” vicious cycle (15). In RA, circRNAs and lncRNAs are involved in the abnormal activation of immune cells, enhanced invasiveness of FLS, and subchondral bone destruction by regulating the spatiotemporal activation of various death modes (such as apoptosis, necroptosis, pyroptosis, autophagy, and ferroptosis).

Traditional Chinese medicine (TCM) has evolved as a distinctive therapeutic paradigm in RA management with over two millennia of documented clinical practice (16, 17). Rooted in the holistic philosophy of “harmonizing Yin-Yang equilibrium” and guided by syndrome differentiation, TCM offers a multi-target intervention strategy through complex herbal formulations. Recent advances in systems biology and network pharmacology are progressively deciphering the “multi-component and multi-target” mechanisms underlying TCM’s therapeutic advantages. Emerging evidence has highlighted the therapeutic potential of TCM in RA through precise modulation of different cell death pathways. TCM compounds exhibit bidirectional regulatory effects on FLS apoptosis, macrophage pyroptosis, and chondrocyte autophagy, thereby restoring immune homeostasis in inflamed joint microenvironment.

This review synthesized the latest evidence elucidating how the regulation of cell death pathways influences RA progression, with an emphasis on crosstalk with ncRNAs. This study aimed to identify possible diagnostic targets for RA and offer a scientific foundation for treating RA using TCM, which will support the advancement of precise diagnostic and individualized treatment strategies for RA.

## Apoptosis and RA

### Overview of apoptosis

Apoptosis, which is another term for PCD, is a well-regulated type of cell death important for the functioning of multicellular organisms (18). Apoptosis pathways are divided into intrinsic and extrinsic types (19). The intrinsic pathway generally involves the mitochondria and is regulated by Bcl-2 family proteins, which consist of both pro-apoptotic and anti-apoptotic members (20). Minor dynamic equilibrium changes can lead to inhibition or promotion of cell death. Conversely, the extrinsic pathway involves death receptors such as the Fas/FasL signaling pathway, which triggers downstream cascades leading to apoptosis (21).

**Figure 2:**
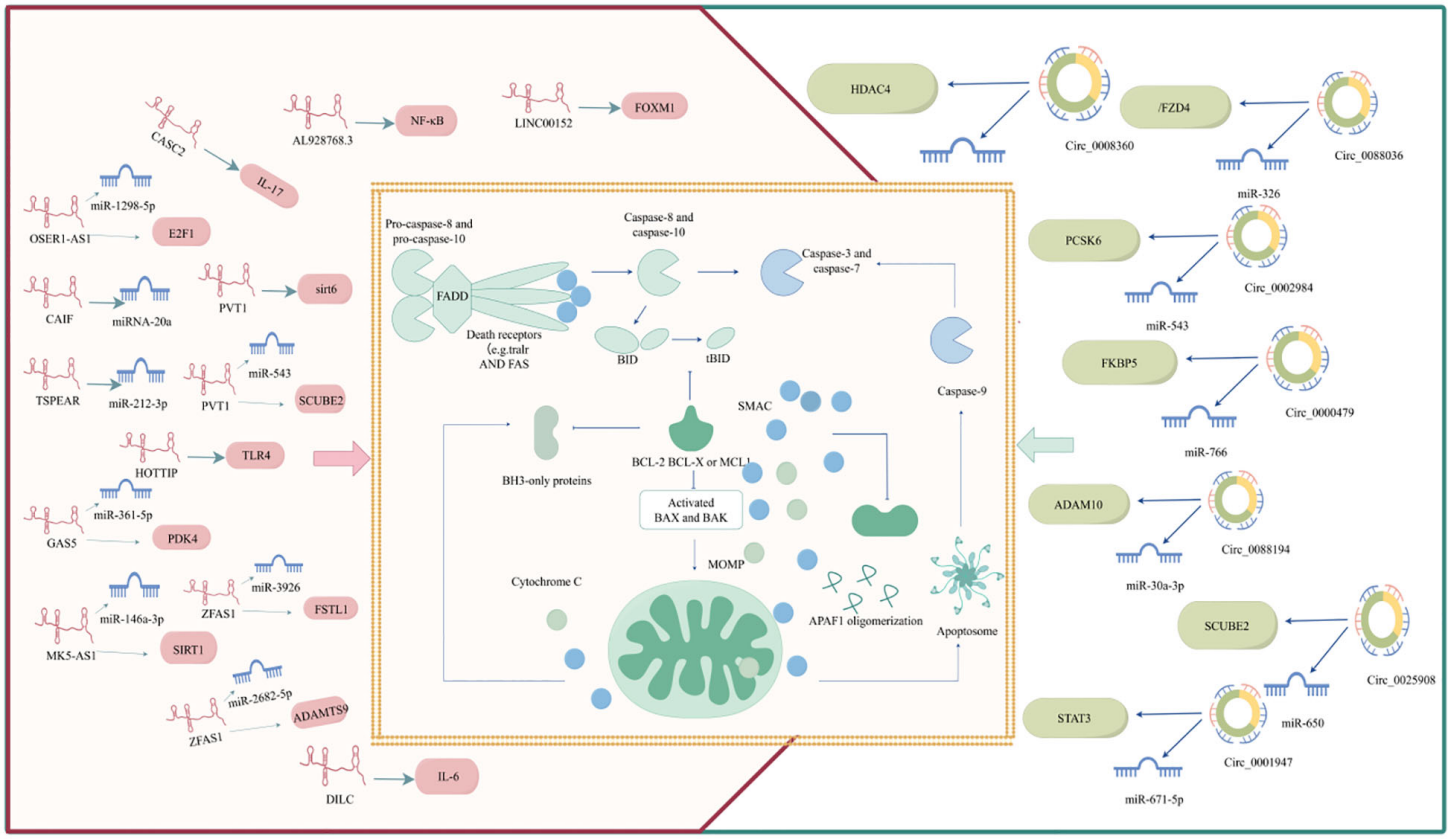
ncRNA regulates RA apoptosis.

Bcl-2 family proteins and caspases (cysteine proteases) play crucial roles in apoptosis pathogenesis. Bcl-2 family proteins control the initiation of apoptosis by regulating mitochondrial membrane permeability, whereas caspases are responsible for the apoptotic process. Apoptosis is initiated by different stimuli that activate internal and external pathways, resulting in a cascade of events (such as the release of apoptotic mediators from the mitochondria and the activation of caspases), all of which are critical in determining cell fate. Apoptosis is a complex and finely regulated process that involves multiple signaling pathways and molecular mechanisms (22). A deeper understanding of apoptosis not only helps to reveal basic cellular functions but may also provide new strategies for RA treatment. RA apoptosis is regulated by ncRNAs. The detailed ncRNA regulation of RA apoptosis is shown in **Figure 2**.

### ncRNAs regulates RA apoptosis

ncRNAs are genes with no coding ability that account for 98% of the human genome and include miRNAs, lncRNAs, and circRNAs (23). LncRNAs refers to RNAs exceeding 200 nucleotides in length, which have the ability to regulate protein-coding gene expression at multiple levels, thereby impacting biological functions, including epigenetic, transcriptional, and post-transcriptional regulatory mechanisms (24). Through back-back splicing, RNA polymerase II transcribes circRNAs that do not have a 5′ cap or a 3′ poly (A) tail, forming covalently closed RNA circles, which results in circRNAs having much greater stability than linear transcripts (25). Considerable evidence has shown that lncRNAs/circRNAs can affect the biological behavior of cells through the ceRNA mechanism. Namely, lncRNAs and circRNAs compete with target mRNA for miRNA attachment, or function as a ‘miRNA sponge,’ indirectly suppressing miRNA by modulating their target mRNA (26). This ceRNA network plays a significant role in controlling apoptosis and regulating RA progression, as shown in **Figure 2**, **Table 1**.

**Table 1:** ncRNA regulates different cell death modalities in RA.

Cell death modalities	NcRNAs	Targets	Cells	Mechanisms
Apoptosis	LncRNA AL928768.3	NF-κB	RA-FLS	Inhibits apoptosis
	LncRNA CASC2	IL-17	RA-FLS	Promotes apoptosis
	LncRNA DILC	IL-6	RA-FLS	Induces apoptosis
	LncRNA TSPEAR	miR-212-3p	RA-FLS	Inhibits apoptosis
	lncRNA CAIF	miRNA-20a	RA-FLS	Supresses apoptosis
	LncRNA OSER1-AS1	miR-1298-5p/E2F1	RA-FLS	Regulates apoptosis
	LINC00152	FOXM1/Wnt/β-catenin	RA-FLS	Regulates apoptosis
	LncRNA HOTTIP	TLR4	RA-FLS	Affects apoptosis
	LncRNA MAPKAPK5-AS1	miR-146a-3p/SIRT1/NF-κB	RA-PBMCs+RA-FLS	Induces apoptosis
	LncRNA ZFAS1	miR-3926/FSTL1	RA-FLS	Promotes apoptosis
	Lnc RNA ZFAS1	miR-2682-5p/ADAMTS9	RA-FLS	Regulates apoptosis
	LncRNA PVT1	sirt6	RA-FLS	Induces apoptosis
	LncRNA PVT1	miR-543/SCUBE2	RA-FLS	Induces apoptosis
	circCBLB	miR-486-5p	RA-FLS	Promotes apoptosis
	Circ-Sirt1	miR-132/Sirt1	MH7A	Induces apoptosis
	Circ_0002984	miR-543/PCSK6	RA-FLS	Inhibits apoptosis
	Circ_0000479	miR-766/FKBP5	RA-FLS	Inhibits apoptosis
	Circ_0088194	miR-30a-3p/ADAM10	RA-FLS	Regulates apoptosis
	Circ_0025908	miR-650/SCUBE2	RA-FLS	Stimulates apoptosis
	Circ_0088036	miR-326/FZD4	RA-FLS	Inhibits apoptosis
	Circ_0001947	miR-671-5p/STAT3	RA-FLS	Inhibits apoptosis
	Circ_0008360	miR-135b-5p/HDAC4	RA-FLS	Promotes apoptosis
Pyroptosis	/	NF-κB/GSDMD	RA-FLS	Alleviates pyroptosis
	/	SMAD2	RA-FLS	Inhibits pyroptosis
	/	HIF-1/BNIP3	RA-FLS	Inhibits pyroptosis
	/	ALOX5	CD4 T cells	Drives pyroptosis
	/	NF-κB/Caspase 3/GSDME	Neutrophil extracellular traps	Induces pyroptosis
	/	Pol β	Macrophage	Induces pyroptosis
	MiR-144-3p	PTEN/PINK1/Parkin	Chondrocytes	Induces pyroptosis
	Circ_0000175	miR-31-5p/GSDME	RA-FLS	Induces pyroptosis
	Circ_0044235	miR-135b-5p/SIRT1	RA-FLS	Regulates pyroptosis
Ferroptosis	/	SIRT1/YY1	RA-FLS	Suppresses ferroptosis
	/	TRPM7/PKCα-NOX4	Chondrocytes	Attenuates ferroptosis
	/	TRPM7/HO-1	Chondrocytes	Inhibits ferroptosis
	/	HMGB1/TLR4/STAT3	M2 macrophages	Inhibits ferroptosis
	/	EZH2	osteoblast-osteoclast	Attenuates ferroptosis
Autophagy	LncRNA MIAT	miR-30a-5p/SOCS1	Macrophages	Promots autophagy
	Lnc RNA ZFAS1	miR-2682-5p/ADAMTS9	RA-FLS	Regulates autophagy
Cell senescence	/	CCN3	RA-FLS	Promotes cell senescence

RA, Rheumatoid arthritis; FLS, fibroblast-like synoviocytes; TLR4, Toll-Like Receptor 4; CCN3, Cell communication network factor 3.

### LncRNAs regulates RA apoptosis

LncRNAs can directly regulate mRNA-mediated apoptosis and participate in RA progression. Sun et al. have highlighted the role of lncRNA AL928768.3 in RA pathogenesis (27). This lncRNA is implicated in promoting the proliferation, invasion, and inflammatory response of FLSs, which are key cellular components involved in RA progression. The mechanism by which lncRNA AL928768.3 exerts its effects involves the activation of the lymphotoxin β-mediated NF-κB signaling pathway, which concurrently inhibits apoptosis in these cells. Another study has highlighted the important role of the lncRNA CASC2 in the pathogenesis of RA (28). Specifically, lncRNA CASC2 was downregulated in the plasma of RA patients, suggesting its involvement in the pathogenesis of RA. Overexpression of CASC2 has been shown to promote apoptosis in FLS by downregulating IL-17(key players in the inflammatory processes of RA and autoimmune disorders) (29). Similarly, Wang et al. have demonstrated that lncRNA DILC is downregulated in the plasma of patients with RA compared to that in healthy controls (30). This downregulation was associated with an increase in IL-6 levels, suggesting an inverse relationship between lncRNA DILC and IL-6 expression in RA patients. Overexpression of lncRNA DILC has been demonstrated to promote the apoptosis of FLS isolated from patients with RA, while also inhibiting the expression of IL-6. This indicates that lncRNA DILC may play a protective role in RA by inducing apoptosis in FLS and reducing IL-6-mediated inflammation. The following two studies have focused on the relationship between lncRNAs and miRNAs in RA apoptosis. TSPEAR-AS2 expression is decreased in RA and it suppresses apoptosis in FLS by reducing miR-212-3p (31). CAIF may inhibit apoptosis in RA-FLS by enhancing the maturation of miR-20a (32). These findings highlight the complex regulatory networks involving lncRNAs and miRNAs in RA, and suggest that targeting these lncRNAs could offer novel therapeutic strategies for regulating cell apoptosis in RA.

In addition, lncRNA OSER1-AS1 is significantly downregulated in the synovial tissue and serum of patients with RA (33). Further mechanistic studies have revealed that lncRNA OSER1-AS1 acts as a ceRNA in RA-FLSs by absorbing miR-1298-5p, leading to increased E2F1 expression, which in turn influences inflammation and apoptosis in RA. Likewise, lncRNA GAS5 hinders RA development by influencing the miR-361-5p/PDK4 axis, which in turn affects FLS proliferation, migration, and apoptosis (34). Moreover, the FOXM1/LINC00152 feedback loop regulates FLS proliferation and apoptosis via the Wnt/β-catenin signaling pathway, demonstrating that these lncRNAs act as ceRNAs that can influence cell apoptosis in RA (35). The transcriptional expression of lncRNAs is regulated by methylation. Toll-like receptor 4 (TLR4) is an important inflammatory mediator in RA-FLS and its expression level is closely related to the intensity of the inflammatory response. LncRNA HOTTIP can influence the methylation state of the TLR4 promoter by interacting with MLL1, resulting in TLR4 promoter methylation, which in turn inhibits RA-FLS growth and triggers cell death and inflammation in RA (36). Another study has focused on RNA methylation of lncRNAs and found that the lncRNA MAPKAPK5-AS1, influenced by m6A, triggers cell death and reduces inflammation by modulating the miR-146a-3p/SIRT1/NF-κB pathway in RA (37). LncRNA ZFAS1 is a newly discovered lncRNA located on chromosome 20q13.13 that plays an oncogenic role in various diseases. Studies have shown that the expression of lncRNA ZFAS1 is markedly increased in synovial tissue and RA-FLS, while its knockdown effectively inhibits the proliferation, migration, and invasion of RA-FLS and reduces inflammatory responses (38). This process may be related to lncRNA ZFAS1 regulating the expression of FSTL1 through miR-3926. A similar study has indicated that the miR-2682-5p/ADAMTS9 axis is modulated by lncRNA ZFAS1, affecting the proliferation, apoptosis, inflammatory response, and autophagy of RA-FLS (39). Plasmacytoma variant translocation 1 (PVT1) is a newly identified lncRNA found on human chromosome 8q24, with 1716 base pairs in length. Evidence suggests that lncRNA PVT1 can bind to the sirt6 promoter to induce sirt6 methylation, thus inhibiting sirt6 transcription, thereby suppressing inflammation and inducing apoptosis in RA-FLS (40). Additionally, lncRNA PVT1 knockdown can induce FLS apoptosis by modulating miR-543-dependent SCUBE2 pathways (41). These studies have indicated that lncRNA ZFAS1 and lncRNA PVT1 play significant roles in RA apoptosis through complex regulatory networks, illustrating that these findings offer a new perspective on potential therapeutic targets for RA.

### CircRNA regulates RA apoptosis

Two studies have shown that circRNAs directly regulate apoptosis in RA patients. One study has shown that circCBLB can inhibit the viability of RA-FLS, increase the apoptosis rate, prolong the cell cycle, and suppress inflammatory reactions (42). Alternatively, the results have shown that circ-Sirt1 can inhibit proliferation, induce apoptosis, and ameliorate inflammation in RA-FLS (43). Additionally, a growing body of research has suggested that circRNAs exert ceRNA regulatory effects on RA. For example, circ_0002984 regulates miR-543 to induce PCSK6 expression in RA, thereby promoting the proliferation and migration of FLSs and increasing the secretion of inflammatory cytokines, while inhibiting apoptosis (44). Similarly, circ_0000479 regulates the expression of FKBP5 by sponge adsorption of miR-766, thereby affecting FLS proliferation, invasion, migration, inflammation, and apoptosis (45).

Numerous studies have highlighted the importance of circRNAs in ceRNA networks related to RA apoptosis, pointing to their potential as new biomarkers and therapeutic targets. For instance, circ_0088194 influences the miR-30a-3p/ADAM10 pathway to enhance proliferation, migration, and inflammation while reducing apoptosis in RA FLSs (46). Inhibition of circ_0025908 inhibits RA-FLS proliferation, migration, invasion, and inflammation while stimulating apoptosis by targeting the miR-650/SCUBE2 axis (47). It has also been shown that circ_0088036 is upregulated in synovial samples from patients with RA (48). By inhibiting circ_0088036, the proliferation of FLSs can be suppressed apoptosis-induced and inflammatory responses are reduced. This process is achieved through the sponging effect of circ_0088036 on miR-326, which in turn regulates the expression of FZD4. Similarly, circ_0001947 promotes the proliferation, inflammatory response, migration, and invasion, and inhibits apoptosis of RA-FLS by modulating the miR-671-5p/STAT3 axis, while overexpression of miR-671-5p can inhibit these processes (49). Apart from the above, Hao et al. have discovered that circ_0008360 suppresses the growth, movement, and inflammation of RA-FLSs while promoting their apoptosis by absorbing miR-135b-5p and increasing HDAC4 levels (50). New strategies for RA treatment can be developed by modulating circRNA expression.

## Pyroptosis and RA

### Overview of pyroptosis

Pyroptosis is a type of PCD akin to apoptosis, characterized by nuclear condensation, chromatin DNA fragmentation, and positive TUNEL staining (51). The creation of pores in the cell membrane during cell death disrupts ion gradient balance, leading to the release of various intracellular elements and proinflammatory mediators. Pyroptosis is mainly driven by gasdermin proteins, specifically GSDMD and GSDME, which create pores in the cell membrane after being cleaved by inflammatory caspases (such as caspase-1, -4, -5, and -11). This process is usually triggered by pathogen-associated molecular patterns (PAMPs) or DAMPs through canonical or non-canonical pathways (52). The canonical pathway involves the activation of inflammasomes (NOD-like receptor family, pyrin domain containing 3 (NLRP3) and AIM2), which recruit caspase-1 to cleave gasdermins and pro-IL-1β/IL-18, whereas the non-canonical pathway directly activates caspase-4/5/11 via intracellular LPS detection (53). Dysregulated pyroptosis contributes to tissue damage and systemic inflammation by amplifying cytokine storms and recruiting the immune cells.

### Pyroptosis involved in the pathogenesis of RA

**Figure 3:**
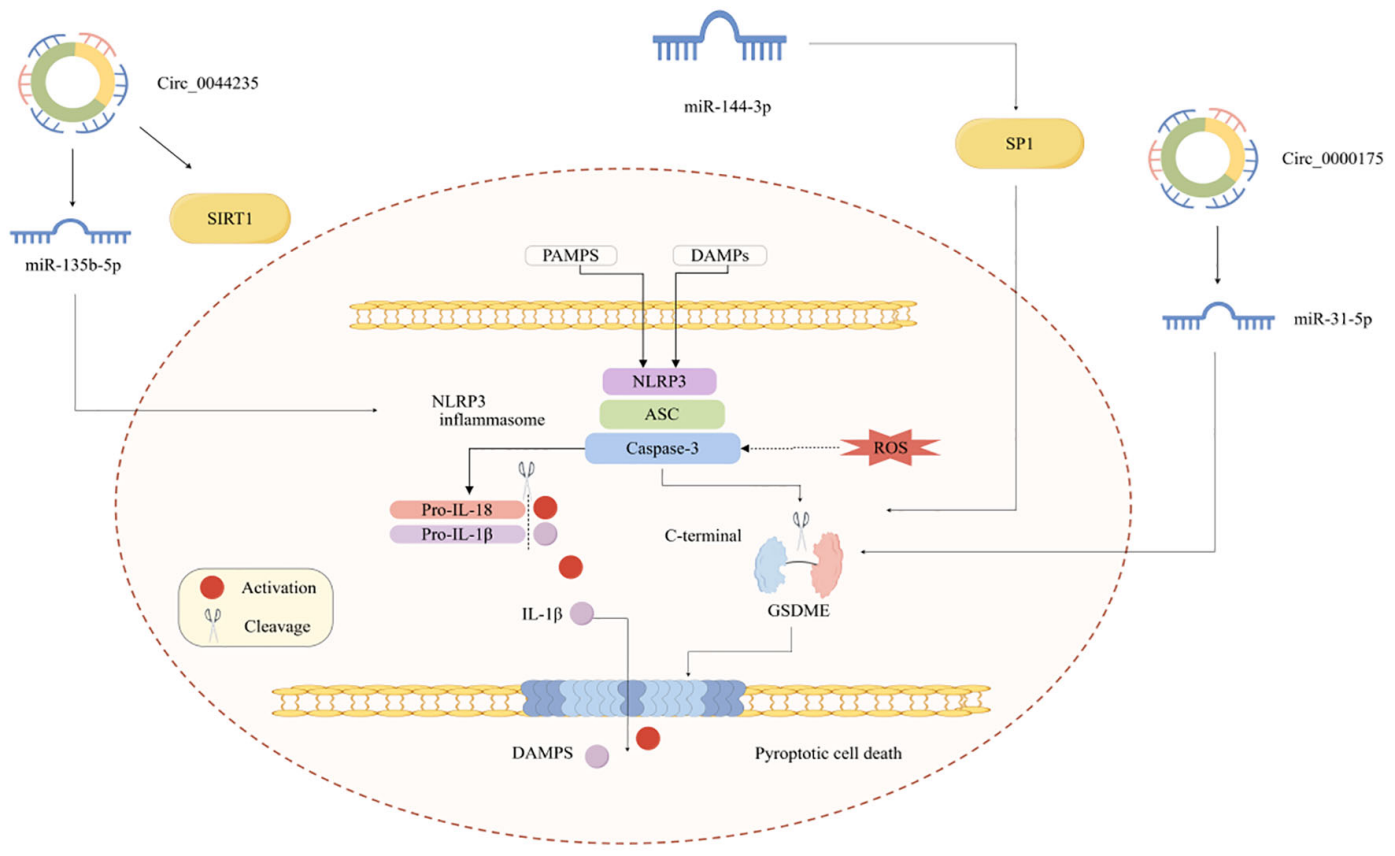
ncRNA regulates RA pyroptosis.

Pyroptosis has also been observed in RA, and its role is increasingly being recognized. However, little is known regarding the role of pyroptosis in RA. Investigating the mechanism of pyroptosis and its connection to RA will enhance our understanding of RA, especially highlighting the role of ncRNAs (**Figure 3**).

Recent single-cell transcriptomic analyses of RA synovial tissue have revealed a pronounced hyperactivation of the NLRP3/caspase-1/GSDMD axis in synovial macrophages, correlating with disease severity and synovial inflammation. This molecular signature distinguishes RA from other inflammatory arthropathies and provides a rationale for targeting pyroptosis in RA therapy (54). Excessive activation of the NLRP3 inflammasome is linked to increased disease activity and severity in RA (55). Evidence has shown that in RA macrophages, the upregulation of NLRP3, caspase-1, and GSDMD suggests that activation of the NLRP3 inflammasome may occur through the activation of caspase-1, which then cleaves GSDMD, leading to cell membrane perforation and the release of cellular contents, thus leading to pyroptosis, an inflammatory form of cell death (56). Similarly, PTX3 and C1q work synergistically by coordinating the overactivation of the NLRP3 inflammasome and inducing GSDMD cleavage, promoting pyroptosis in RA serum-pre-incubated monocytes (57). Interestingly, IL-37 is a novel anti-inflammatory cytokine that can reduce pyroptosis in TNF-α-induced FLS by suppressing the NF-κB/GSDMD signaling pathway (58). Another investigation has revealed that SMAD2 can prevent pyroptosis in RA-FLS by lowering the expression of important parts of the NLRP3 inflammasome complex, including NLRP3, ASC, and caspase-1 (59). This inhibitory effect not only reduces the secretion of inflammatory factors but also alleviates RA symptoms through the TGF-β signaling pathway. Exploring the mechanisms linking inflammation and pyroptosis is of great significance in RA research. A study has discovered that LPS can cause pyroptosis in synovial fibroblasts through the NLRP3/caspase-1/gasdermin D and caspase-3/gasdermin E pathways, which support each other (60).

m6A methylation changes NLRP3, which is related to the regulation of apoptosis and inflammation. In RA, WTAP, a key m6A methyltransferase, promotes pyroptosis in fibroblast-like synovial cells by upregulating m6A modification of NLRP3 (61). Autophagy and pyroptosis in RA interact in a complex process involving multiple signaling pathways and cellular mechanisms. Previous studies have shown that the HIF-1/BNIP3 pathway plays a crucial role in this process (62). Specifically, HIF-1 promotes mitochondrial autophagy by regulating BNIP3, thereby inhibiting hypoxia-induced pyroptosis in RA-FLSs. The pathogenesis of RA involves complex interactions between multiple cell types and pyroptosis. For example, pyroptosis of CD4+ T cells is a critical step in the ALOX5 gene, which is believed to play a significant role in driving pyroptosis and tissue inflammation in CD4+ T cells during RA (63). Additionally, NETs can induce apoptosis in RA FLSs. Studies have shown that NETs can induce pyroptosis in RA-FLSs and facilitate phenotypic transformation by targeting the NF-κB/caspase 3/GSDME axis (64). Macrophage pyroptosis, a type of cell death that promotes inflammation, plays a crucial role in RA, and its regulation is accomplished by suppressing macrophage pyroptosis (65). Furthermore, there is still a lack of research on the mechanisms underlying RA chondrocyte pyroptosis. A single study has demonstrated that miR-144-3p can enhance IL-1β-induced pyroptosis in RA chondrocytes by downregulating PTEN expression and hindering the PINK1/Parkin signaling pathway (66).

Research has increasingly suggested that circRNA-mediated ceRNA networks regulate pyroptosis. It has been shown that circ_0000175 level is increased; mechanistically, circ_0000175 can promote pyroptosis and initiate an inflammatory response in RA via the miR-31-5p/GSDME pathway (67). Another study by Chen et al. has indicated that hsa_circ_0044235 influences NLRP3-driven pyroptosis via the miR-135b-5p-SIRT1 pathway to control RA progression (68).

Collectively, pyroptosis has significant research value in the pathogenesis of RA. By delving into the mechanisms of RA, new targets and strategies for its treatment can be identified. Future studies should focus on the regulation of pyroptosis to reduce inflammatory responses and tissue damage in RA, thereby improving patients’ clinical symptoms and quality of life.

## Ferroptosis and RA

### Overview of ferroptosis

Ferroptosis is a type of regulated cell death that relies on iron, marked by the deadly buildup of lipid peroxides, setting it apart from apoptosis, necrosis, and autophagy in both biochemical and structural aspects (69). Mechanistically, ferroptosis is driven by failure of the glutathione (GSH)-glutathione peroxidase 4 (GPX4) antioxidant axis, where GPX4 inactivation or depletion prevents the reduction of lipid hydroperoxides, leading to membrane destabilization. This process is tightly linked to cellular iron metabolism, as excess labile iron catalyzes lipid peroxidation via Fenton reactions and activates iron-containing enzymes that propagate oxidative damage. In a recent cohort study of RA patients, a marked downregulation of GPX4 in peripheral blood mononuclear cells is identified as a key ferroptosis marker. This decrease in GPX4 levels is significantly correlated with RA disease activity, supporting a central role for ferroptosis in RA pathogenesis (70). Additionally, dysregulation of cystine uptake through the system Xc^−^ transporter, which provides cysteine for GSH synthesis and exacerbates the redox imbalance (71, 72). p53, a crucial tumor suppressor gene, plays a key role in ferroptosis. p53 influences intracellular iron levels by regulating the expression of iron metabolism-related genes, thereby controlling the onset of ferroptosis (73). Ferroptosis Inhibitor 1 (FSP1) is a key anti-ferroptotic factor that reduces coenzyme Q10 to coenzyme Q10 alcohol through the action of NAD(P)H-coenzyme Q10 oxidoreductase, thereby capturing lipid peroxyl radicals and inhibiting lipid peroxidation reactions, which in turn suppresses ferroptosis. Iron metabolism plays a crucial role in ferroptosis (74). An overabundance of iron can cause a rise in ROS levels inside cells, which in turn encourages lipid peroxidation and triggers ferroptosis. The regulation of iron metabolism involves multiple proteins and pathways, including iron transporters, storage proteins, and excretion proteins (75).

Emerging evidence has highlighted a bidirectional interplay between ferroptosis and inflammation, wherein inflammatory signaling exacerbates ferroptotic susceptibility, whereas ferroptotic cell death amplifies inflammatory cascades through DAMPs (76, 77). Pro-inflammatory cytokines (such as TNF-α, IL-6, and IL-1β) modulate ferroptosis by suppressing system Xc^−^-mediated cystine uptake or upregulating iron-regulatory proteins, thereby depleting GSH and increasing labile iron pools (78). Conversely, ferroptosis-derived lipid peroxidation products and released intracellular components act as DAMPs to activate pattern recognition receptors (PRRs) on macrophages and dendritic cells, triggering the NF-κB and NLRP3 inflammasome pathways to propagate IL-1β and IL-18 secretion. Notably, iron overload in ferroptotic cells potentiates inflammasome activation via mitochondrial ROS (mtROS)-dependent mechanisms, thereby establishing a feedforward loop between ferroptosis and pyroptosis.

### Ferroptosis involved in the pathogenesis of RA

Based on the GEO database, Cai et al. have found that MMP13 and GABARAPL1, as markers of ferroptosis, are closely related to oxidative stress and immune regulation, and have important potential applications in the diagnosis and treatment of RA (79). Another study has utilized bioinformatics analysis of the GEO database (accession number: GSE12021) and identified EGR1 and CDKN1A as hub ferroptosis-related genes in RA pathogenesis. In similar studies, GABARPL1, DUSP1, JUN, and MAPK8 are downregulated and act as diagnostic biomarkers and therapeutic targets for RA by regulating ferroptosis (80). Similarly, He et al. have discovered and confirmed PTGS2, ENO1, and GRN as possible biomarkers related to RA ferroptosis (81). Mechanistically, a high level of ENO1 expression was found to inhibit ACO1, which reduces ferroptosis in proliferating RA-FLS. These newly identified molecules are not only important biomarkers of ferroptosis in RA, but have important research and clinical applications. In addition, Fan et al. have acquired FRGs from Ferroptosis database (FerrDb); VEGFA, PTGS2, and JUN might be the key genes related to ferroptosis in RA, primarily participating in the FoxO signaling pathway (82). Xia et al. have conducted an enrichment analysis on 29 differentially expressed ferroptosis-related genes in RA and found that these genes may be involved in FoxO signaling pathways and genetic metabolic disorders (83). Among the concerns is that RRM2 has been identified as a biomarker of high diagnostic value, which may provide new evidences for the clinical transformation (84). SLC2A3 predominantly encodes neuronal GLUT3, which has been identified as an important potential biomarker for RA ferroptosis (85). Further studies have confirmed that RSL3 can induce ferroptosis in RA-FLS by downregulating SLC2A3. SIRT1’s action mechanism in RA is complex and involves multiple signaling pathways and cellular processes. Research has indicated that YY1 transcriptionally suppresses SIRT1, which in turn inhibits ferroptosis in RA-FLS, potentially offering new diagnostic and therapeutic targets for RA (86).

RA pathogenesis involves interactions between multiple cell ferroptosis and inflammatory mediators. Chondrocyte ferroptosis is involved in RA development. Research has indicated that TRPM7 reduces damage to RA articular cartilage and chondrocyte ferroptosis through the PKCα-NOX4 pathway (87, 88). A different study has found that inhibiting TRPM7 alleviates damage to RA articular cartilage and chondrocyte ferroptosis through the TRPM7/HO-1 pathway (89). Thus, the mechanism of action of TRPM7 in RA is closely related to chondrocyte ferroptosis. An in-depth study of the molecular mechanism of TRPM7 in ferroptosis will provide new targets and strategies for RA. The susceptibility of M2 macrophages to ferroptosis is an important research area in RA. According to Feng et al., the HMGB1/TLR4/STAT3 pathway is essential for ferroptotic M2 macrophages to worsen synovial inflammation in patients with RA. In RA, the equilibrium between osteoblasts and osteoclasts is disturbed, resulting in bone loss and destruction (90). EZH2 is an epigenetic regulatory factor that plays a crucial role in the pathophysiology of RA. Recent research has shown that sh-EZH2 reduces the clinical symptom score in CIA mice, alleviates systemic inflammation (TNF-α and IL-6), and inhibits bone ferroptosis, and partially reestablishes the balance between osteoblasts and osteoclasts, as indicated by immunohistochemical staining of associated markers (91). These findings not only enhance our understanding of RA pathophysiology but also provide novel therapeutic targets for developing precision medicine approaches against ferroptosis-driven joint destruction.

## Autophagy and RA

### Overview of autophagy

Autophagy is a crucial lysosome-based degradation mechanism that helps maintain cellular balance by removing damaged organelles, misfolded proteins, and intracellular pathogens (91). Mechanistically, autophagy is orchestrated by a cascade of autophagy-related (ATG) proteins that coordinate the formation of double-membrane autophagosomes, which subsequently fuse with lysosomes for cargo degradation (92). Key regulators include the mTOR-ULK1 signaling axis, which integrates nutrient and energy status, and the Beclin-1/VPS34 complex, which governs autophagosome nucleation. Autophagy dysregulation has emerged as a critical pathogenic mechanism in diverse diseases.

Autophagy and inflammation exhibit dynamic and context-dependent interplay that significantly influences disease occurrence and development (93). As a homeostatic mechanism, autophagy suppresses excessive inflammation by sequestering and degrading endogenous danger signals, such as damaged mitochondria, releasing ROS, and mtDNA, which otherwise activate the NLRP3 inflammasome and drive IL-1β/IL-18 maturation (94). Conversely, autophagy deficiency in immune cells (such as macrophages) leads to p62/SQSTM1 accumulation, which aberrantly activates NF-κB and exacerbates pro-inflammatory cytokine production. Paradoxically, autophagy also facilitates inflammation resolution by clearing inflammasomes and degrading pro-IL-1β via autophagosome-lysosome trafficking (95).

### Autophagy involved in the pathogenesis of RA

LncRNAs play a vital role in epigenetic regulation and are involved in controlling autophagy through different mechanisms. Recent findings have underscored the significant impact of lncRNAs in regulating autophagy, thereby affecting RA (96). Dysregulated lncRNAs act as epigenetic regulators or ceRNAs to fine-tune autophagy-related signaling pathways, thereby contributing to synovial hyperplasia, immune dysregulation, and joint destruction.

Shang et al. have revealed that autophagy-related lncRNAs exhibit significant changes in expression in RA (97). Among these, the expression level of ENST0000584721.1 is significantly upregulated, while ENST0000615939.1 shows a downward trend. The results indicated that autophagy-associated lncRNAs are vital in the development of RA. Wen et al. have investigated autophagy-associated lncRNAs in the PBMCs of patients with RA by high-throughput sequencing. They have found that MIR22HG, DSCR9, LINC01189, MAPKAPK5-AS1, and ENST00000619282 can serve as potential biomarkers for RA (98). Further correlation analysis revealed that these lncRNAs were related to the clinical indices and self-perception of the patients. In addition, another study has identified lncRNA expression profiles in exosomes derived from the synovial fluid of RA patients, which show significant enrichment of “autophagy” (99). They have concluded that ENST00000433825.1 is highly expressed in RA and is positively correlated with CRP. Collectively, these studies reveal the significant role of autophagy-related lncRNAs and genes in RA, providing new insights for future diagnosis and treatment.

The lncRNA, MIAT, plays a crucial role in RA, particularly in inflammatory responses and autophagy. Studies have demonstrated that lncRNA MIAT serves as a ceRNA, sequesters miR-30a-5p, and negatively impacts SOCS1, leading to decreased inflammation and autophagy in RA macrophages (100). This discovery not only broadens our understanding of MIAT’s role of MIAT in inflammation and autophagy but also provides new insights for future clinical interventions for RA.

## Cell senescence and RA

### Overview of cell senescence

Cellular senescence refers to a permanent halt in the cell cycle caused by various stress factors (such as DNA damage, telomere shortening, oncogene activation, and oxidative stress) (101). It is characterized by persistent proliferative arrest (via G1/S or G2/M phase blockade), metabolic reprogramming, and senescence-associated secretory phenotype (SASP), which releases pro-inflammatory cytokines (e.g., IL-6, IL-8), proteases, and growth factors to remodel the microenvironment (102). Mechanistically, senescence is orchestrated by the p53-p21 and p16INK4a/Rb pathways, which inhibit cyclin-dependent kinases (CDKs) to block cell cycle progression (103). The SASP is regulated by NF-κB, mTOR, and AMPK, which amplify inflammation and tissue dysfunction. Accumulated senescent cells exacerbate chronic inflammation and tissue degeneration via SASP-mediated bystander effects. Key molecules [such as p16INK4a/Rb, p21, and SASP components (MMPs and HMGB1)] serve as biomarkers and therapeutic targets. Emerging strategies, including senolytics (such as dasatinib/quercetin) and SASP inhibitors, aim to selectively eliminate senescent cells or modulate their secretory profiles, offering promise for treating senescence-associated pathologies (104, 105).

Emerging evidence has indicated that the dynamic crosstalk between inflammation and cellular senescence is complex and mutually influential, forming a vicious cycle (106). During cellular senescence, the immune system undergoes remodeling, leading to decreased immune function and the emergence of chronic inflammatory states, phenomena known as immune senescence (immunosenescence) and inflammatory senescence (inflammaging) (107, 108). In this process, the inflammatory response further accelerates the aging process. In addition, chronic inflammation is not only a result of aging, but also a driving factor. At the same time, during cellular senescence, cells secrete large amounts of pro-inflammatory factors, forming the so-called SASP, which not only exacerbates local inflammatory responses but can also affect the entire body through the circulatory system, leading to systemic inflammation (109). Therefore, the interaction between inflammation and aging is a complex process that involves the interaction of multiple cytokines and signaling pathways. Deciphering the cell type-specific senescence dynamics and their crosstalk with inflammatory networks will advance precision therapeutics for various diseases.

### Cell senescence involved in the pathogenesis of RA

RA pathogenesis is closely linked to cellular senescence. Ao et al. have systematically analyzed senescence-associated genes from the CellAge database using an integrated bioinformatics approach (110). Through two distinct machine learning algorithms (random forest and LASSO Cox regression), they have successfully identified three pivotal senescence-associated genes (DHX9, CYR61, and ITGB) that exhibit strong prognostic relevance in RA pathogenesis. Notably, these biomarkers demonstrate significant functional enrichment in the immune cell infiltration processes. In a complementary multi-omics investigation using the HAGR database, Xu and colleagues have conducted a systematic study to identify 6 senescence drivers linked to RA (IL-6, IL7R, IL2RG, CDK1, PTGS2, and LEP) using differential expression and protein-protein interaction network analyses (111). The analysis of immune infiltration showed a strong positive relationship between these genes and the presence of M1 macrophages. Laboratory experiments have shown that IL-6 is a dependable indicator for diagnosing and treating RA. In the disease progression of RA, macrophages act as more than just inflammatory mediators; they also interact with FLS within the joint, forming a complex cellular network that maintains and exacerbates the local inflammatory environment by secreting various cytokines and chemokines. Studies have shown that aged macrophages trigger pro-inflammatory programs, thereby promoting the progression of RA, particularly an increase in M1-type senescence macrophages (112).

Senescence of T cells is an important area of research in RA. Studies have shown that CD56^+^ T cells with features of senescence are expanded in RA, which is considered a potential target for therapy (113). Cell communication network factor 3 (CCN3) plays a crucial role in the pathogenesis of RA. One of its mechanisms is the promotion of senescence in joint cells and the generation of osteoclasts. Understanding the molecular mechanisms of CCN3 is important for developing new therapeutic strategies (114). Synovial stem cells typically have the ability to modulate immune responses during the pathological process of RA. Synovial mesenchymal stem cells exhibit impaired immune regulatory functions in a chronic inflammatory environment in patients with RA (115). This impairment may be related to the continuous stimulation by inflammatory factors and induced cell senescence, leading to a decrease in the immunosuppressive capacity of MSCs, thereby exacerbating RA progression.

Cell senescence plays a complex role and holds significant research value in the pathogenesis of RA as it may influence the progression and pathological characteristics of RA through multiple pathways. Investigating cellular senescence and its associated mechanisms may help provide new insights and directions for RA diagnosis and treatment.

## Necroptosis and RA

### Overview of necroptosis

Necroptosis is a regulated form of PCD that shares morphological features with necrosis but is mechanistically distinct from apoptosis (116). It is characterized by cellular swelling, plasma membrane rupture, and release of intracellular contents, which triggers inflammatory responses (117). This cell death pathway is typically initiated when apoptosis is inhibited, such as during viral infection or caspase-8 dysfunction, and involves receptor-interacting protein kinase 1 (RIPK1), RIPK3, and mixed-lineage kinase domain-like pseudokinase (MLKL). Upon activation (e.g. by TNF receptor family members, TLRs, or pathogen sensors), RIPK1 recruits and phosphorylates RIPK3 to form a necrosome complex. RIPK3 subsequently phosphorylates MLKL, inducing its oligomerization and translocation to the plasma membrane where it disrupts membrane integrity and leads to cell lysis (118). Recent clinical biomarker analyses have established that elevated expression and activation of RIPK1 in synovial fibroblasts is closely associated with radiographic joint erosion and disease progression in RA patients. These findings highlight the pathophysiological importance of necroptosis in the synovial microenvironment and its potential as a therapeutic target (119). Beyond its canonical RIPK1-RIPK3-MLKL axis, necroptosis intersects innate and adaptive immune signaling pathways, amplifying or dampening disease progression through context-dependent mechanisms (120).

Recent studies have revealed that necroptotic cells release DAMPs such as HMGB1 and mitochondrial DNA, activating pattern recognition receptors (e.g., TLRs and NLRP3 inflammasomes) to drive the inflammatory response (121). This immunogenic cell death modality bridges the tissue damage via systemic immune responses. Defective clearance of necroptotic debris exacerbates autoantibody production via sustained DAMP exposure in autoimmune disorders such as systemic lupus erythematosus (SLE).

### Necroptosis involved in the pathogenesis of RA

Necroptosis plays a crucial role in the pathophysiology of RA, which has garnered significant attention in recent studies of rheumatic diseases. In RA, cell death is not only a result of inflammation but may also be a cause. The loss of cell membrane integrity leads to the release of DAMPs, which are typically sequestered within cells. The release of DAMPs enhances local inflammation and stimulates the production of cytokines and chemokines, thus influencing innate immune responses. Uncontrolled or excessive cell death is thought to contribute to chronic inflammation in RA.

By employing three machine learning algorithms (LASSO, random forest, and SVM-RFE), Wan et al. have also identified 5 core necroptosis-related genes (FAS, CYBB, TNFSF10, EIF2AK2, and BIRC2) in RA that demonstrated significant associations with immune cell infiltration, particularly macrophages (122). These molecular targets show promising potential for clinical diagnostic applications and immunotherapeutic development in RA management. Notably, a parallel investigation has demonstrated that FAS, MAPK8, and TNFSF10 emerge as pivotal regulators of necroptosis, potentially contributing to immune microenvironment remodeling in RA through their interactions with the ceRNA network (123). Another seminal study has identified FUS, TRA2B, EEF2, CPSF6, and STAT3 as necroptosis-associated genes with significant diagnostic value in RA that are closely linked to immune cell infiltration dynamics (124). Furthermore, necroptosis in macrophages plays a crucial role in the progression of RA. It has been found that TNF-α can initiate necroptosis in macrophages, which promotes the release of inflammatory factors like 14-3-3η, thereby aggravating inflammation and tissue damage (125). Interleukin-18-binding protein (IL-18BP), a natural inhibitor of IL-18, modulates biological activity by binding to IL-18 and preventing its binding to receptors. Studies have shown that IL-18BP enhances the apoptosis of FLS while reducing necroptosis in FLS/chondrocytes and apoptosis in chondrocytes, indicating its potential to preserve joint health (126). Moreover, the RIP1 inhibitor Necrostatin-1 has shown potential in anti-necrotic apoptosis in various disease models. Studies have demonstrated that Nec-1 can ameliorate damage to articular chondrocytes in adjuvant-induced arthritis rats by inhibiting ASIC1a-mediated necrotic apoptosis (127). This process might be linked to Nec-1’s suppression of crucial proteins in the necrotic apoptosis pathway.

Overall, the study of necroptosis in RA not only helps us understand its pathophysiological mechanisms but also provides potential targets for developing new therapeutic strategies. By delving into the signaling pathways of necroptosis, we may discover new methods to inhibit cell death, thereby offering more effective treatment options for RA patients.

### Potential TCM treats RA by regulating different cell death modalities

TCM has unique advantages in the treatment of RA, primarily owing to its holistic approach and syndrome differentiation (128). TCM regulates the body’s balance through multi-component and multi-target methods, improves symptoms, and reduces inflammatory responses (129). Guided by the philosophy of holism, TCM emphasizes the dynamic balance of the entire organism, rather than focusing solely on localized joint lesions (130, 131). It systematically regulates immune dysfunction, mitigates inflammatory cascades, and inhibits bone destruction through multi-component interactions. Simultaneously, the principle of “Bianzheng Lunzhi” (syndrome differentiation and treatment) enables personalized therapeutic strategies tailored to individual patterns (such as cold-damp obstruction, damp-heat accumulation, or blood stasis) through precise herb combinations and dosage adjustments. This characteristic integration of systemic regulation and individualized therapy not only ameliorates clinical symptoms but also improves patients’ long-term quality of life and provides new ideas and methods for modern medicine. TCM exerts therapeutic effects on RA by regulating different cell death modalities through ncRNAs (**Table 2**).

**Table 2:** Potential TCM treats RA by regulating different cell death modalities.

Cell death modalities	TCM	Targets	Mechanisms
Apoptosis	Xinfeng capsule	lncRNA MAPKAPK5-AS1	Promotes apoptosis and attenuates inflammation
	Xinfeng capsule	circ-CBLB	Promotes apoptosis and inhibits proliferation
	Triptolide	lncRNA ENST00000619282	Promotes apoptosis and attenuates inflammation
	Tanshinone IIA	lncRNA GAS5	Promotes apoptosis
	Quercetin	lncRNA MALAT1	Promotes apoptosis
	Paeoniflorin	lncRNA MALAT1	Inhibits Wnt1/β-catenin pathway and promotes apoptosis
Pyroptosis	Bitongqing	NLRP3/Caspase-1/GSDMD	Suppresses macrophage pyroptosis
	Xinfeng capsule	NLRP3/GSDMD	Inhibits pyroptosis
	Yiqi Yangyin Tongluo	ASIC1a/NLRP	Attenuates pyroptosis
	Jinwujiangu capsule	NLRP3/CAPSES/GSDMD	Inhibits pyroptosis
	Punicalagin	NF-κB signaling pathway	Downregulates M1 macrophage and pyroptosis
	Periplogenin	NLRP3/Caspase-1/GSDMD	Inhibits pyroptosis
	Mangiferin and cinnamic acid	TLR4/NFκB/NLRP3	Inhibits pyroptosis
Ferroptosis	Jinwu Jiangu capsule	SLC7A11/GSH/GPX4	Induces ferroptosis
	Amentoflavone	PIN1	Induces ferroptosis to alleviate proliferation, migration, invasion and inflammation
	Asiatic acid	Nrf2/HMOX1	Induces ferroptosis to relieve inflammation

NLRP3, NOD-like receptor family, pyrin domain containing 3; TLR4, Toll-Like Receptor 4; GSH, glutathione; GPX4, glutathione peroxidase 4.

Xinfeng capsule (Approval No.: Wanyao Zhizi Z20050062, Patent No.: ZL 2013 1 0011369.8), a hospital-prepared formulation from the First Affiliated Hospital of the Anhui University of Chinese Medicine, exemplifies the clinical translation of TCM principles for RA management (132, 133). Composed of Astragalus membranaceus (Huangqi), Coix lacryma-jobi (Yiyiren), Tripterygium wilfordii (Leigongteng), and Scolopendra subspinipes (Wugong), it synergistically embodies the therapeutic strategy of “strengthening the spleen, resolving dampness, and unblocking collaterals to alleviate impediment” (134). A previous study has indicated that Xinfeng capsules enhance apoptosis and reduce inflammation in RA-FLS by modulating lncRNA MAPKAPK5-AS1 (135). A different study has found that Xinfeng capsules can reduce proliferation and inflammation and enhance apoptosis through the upregulation of circ-CBLB (136). Importantly, recent meta-analyses and clinical trials have provided robust evidence for the clinical efficacy of TCM interventions in RA. A randomized controlled trial conducted in 2023 has demonstrated that astragaloside IV, when combined with methotrexate, can significantly improve clinical outcomes and reduce inflammatory markers in RA patients compared to methotrexate monotherapy. These findings substantiate the value of TCM as an adjunct therapy in RA management (137). Baicalein, another Astragalus membranaceus-derived compound, has been shown to stabilize circRNA_0000396 by targeting m6A methyltransferase, further influencing ferroptosis and pyroptosis networks (138).

Triptolide, a diterpene triepoxide derived from the Chinese herb Tripterygium wilfordii Hook F, has been utilized in TCM to address inflammation and autoimmune disorders (139). One such lncRNA, ENST00000619282, has been shown to be implicated in the regulation of apoptosis and inflammation in RA-FLS. Triptolide downregulates this lncRNA, thereby promoting apoptosis and attenuating inflammation in RA-FLS (140). According to recent mechanistic studies, triptolide can epigenetically modulate DNA methylation patterns, thereby regulating the transcription of lncRNA NEAT1, which in turn affects downstream apoptotic and inflammatory pathways in RA synoviocytes (141). Tanshinone IIA, a significant lipophilic compound derived from the roots of Salvia miltiorrhiza Bunge (also known as danshen), has various anti-inflammatory and antioxidative effects. Li et al. have demonstrated that Tanshinone IIA promotes RA-FLS apoptosis and inhibits PI3K/AKT signaling by upregulating the lncRNA GAS5 (142). Quercetin is a flavonoid found in many plants and possesses various pharmacological benefits, including antioxidant, anti-inflammatory, and anti-apoptotic properties. A previous study has indicated that quercetin promotes RA-FLS apoptosis and inhibits the activation of the PI3K/AKT pathway by upregulating lncRNA MALAT1 (143). Similarly, paeoniflorin, sourced from the traditional Chinese herb Paeonia lactiflora, functions as a monoterpene glycoside with anti-inflammatory and anti-apoptotic actions. It has been suggested that paeoniflorin inhibits the Wnt1/β-catenin pathway and promotes the apoptosis of RA-FLS by upregulating lncRNA MALAT1 (144). These findings collectively demonstrate that TCM exerts pro-apoptotic effects on RA synovial cells through the modulation of ncRNA networks, providing scientific evidence to support the clinical application of TCM in treating RA.

The TCM prescription, Bitongqing (BTQ), exhibits significant efficacy in the clinical treatment of RA. In CIA rats, its mechanism of action might involve inhibiting macrophage pyroptosis and modulating the NLRP3/Caspase-1/GSDMD signaling pathway (145). Specifically, BTQ effectively reduces the release of inflammatory factors by inhibiting the activation of NLRP3 inflammasomes, thereby alleviating RA inflammatory responses and tissue damage. Similarly, Xinfeng capsules have similar anti-RA effects. Wang et al. have shown that Xinfeng capsules alleviate the local inflammatory response of joints in RA, possibly by inhibiting pyroptosis of the FLS via inhibition of the NLRP3/GSDMD pathway (146). Additionally, the activation of ASIC1a increases intracellular calcium concentration, thereby activating NLRP3 inflammasomes, resulting in cell apoptosis and the release of inflammatory factors, which are involved in the pathological process of RA. Studies have shown that the Yiqi Yangyin Tongluo method to unblock meridians can effectively reduce RA chondrocyte apoptosis by regulating the ASIC1a/NLRP3 signaling pathway (147). Jinwujiangu Capsule is a TCM formulation that has shown promise in treating RA by targeting the FLS. The action mechanism involves the inhibition of RA-FLS pyroptosis through the NLRP3/CAPSES/GSDMD pathway (148). Further research on the specific mechanisms and clinical efficacy of this treatment may pave the way for developing new therapeutic options for managing RA. Punicalagin (PUN), derived from pomegranate peel, can prevent joint inflammation, cartilage damage, and widespread bone destruction in CIA mice (149). The specific mechanism involves inhibition of M1 phenotype polarization and pyroptosis. Periplogenin (PPN) is a natural compound that acts as a cardiac glycoside and provides anti-inflammatory and pain-relieving benefits in different conditions. The main underlying mechanism of PPN anti-RA in inhibiting pyroptosis and improving inflammation is by regulating the NLRP3/Caspase-1/GSDMD signaling pathway (150). Simultaneously, research has indicated that a new drug combination of Mangiferin and Cinnamic Acid could effectively alleviate RA by targeting and inhibiting the TLR4/NFκB/NLRP3 activation-induced pyroptosis pathway (151). These results suggest that this combination has great potential for further development and clinical applications in RA.

In recent years, TCM has made significant progress in the treatment of RA via the ferroptosis pathway. This is mainly due to that ferroptosis is linked to the inflammatory response in RA, while TCM has anti-inflammatory properties. According to Ling et al., Jinwu Jiangu capsules can influence lipid metabolism to reduce RA inflammation by decreasing lipids linked to ferroptosis, mainly via the coordinated regulation of the SLC7A11/GSH/GPX4 pathway in M1 macrophages (152). These findings provide an innovative therapeutic basis for RA treatment and expand the clinical application of Jinwu Jiangu capsules. Amentoflavone (AMF), a polyphenolic compound obtained from Selaginella tamariscina extracts, has been shown to reduce proliferation, migration, invasion, and inflammation in RA-FLS (153). This is mainly achieved by the suppression of PIN1 inducing ferroptosis. In addition, the anti-RA effects of asiatic acid has also been investigated. Specifically, asiatic acid can effectively relieve inflammation in CIA model rats by promoting Fe^2+^ accumulation via the Nrf2-HMOX1 pathway (154). These studies indicate that the development of TCM with significant progress in the field of rheumatic disease treatment provides potential for TCM treatment of RA by inducing cell ferroptosis.

## Conclusions and perspectives

In this review, we systematically summarized the roles of diverse cell death modalities (including apoptosis, autophagy, ferroptosis, necroptosis, cell senescence, and pyroptosisin) the pathogenesis of RA, with a focus on their regulatory effects on synovial cells, macrophages, chondrocytes, and T cells. Notably, we highlighted the emerging significance of ncRNAs as critical regulators of these processes. Through mechanisms such as sponging miRNAs or forming ceRNA networks, ncRNAs intricately modulate cell death pathways, thereby influencing RA progression and immune-inflammatory imbalances. Furthermore, the therapeutic potential of TCM in RA management was discussed. Both TCM compounds and monomers have demonstrated efficacy in regulating cell death pathways. These agents often act via multi-target mechanisms, synergistically restoring cellular homeostasis and alleviating RA-related pathological damage.

Although this review provides critical insights into cell death mechanisms in RA, there are still some limitations and unresolved challenges. First, crosstalk between distinct cell death modalities (such as apoptosis and senescence, autophagy, and ferroptosis) remains underexplored. Such interactions may drive RA progression synergistically or antagonistically; however, their molecular underpinnings and spatiotemporal dynamics in synovial microenvironments remain poorly defined. Second, the heterogeneity of cell death responses across different RA-associated cell types (synovial fibroblasts versus macrophages) requires further investigation. Variations in death susceptibility and signaling crosstalk among these cells likely contribute to disease heterogeneity. However, this is rarely addressed in current studies. Third, despite promising preclinical findings, clinical translation remains hindered by insufficient evidence from large-scale, multi-center, and randomized controlled trials (RCTs). Most existing studies rely on small cohorts or animal models, limiting generalizability and failing to account for patient-specific variables such as genetic background and comorbidities. Fourth, although TCM exhibits multi-target therapeutic potential, its broader application is constrained by the inconsistent quality control of herbal formulations and incomplete mechanistic characterization. Validating TCM-derived compounds through rigorous pharmacokinetic and clinical studies to bridge traditional knowledge and modern precision medicine. Integrating multi-omics approaches and advanced disease models will deepen our understanding of the RA pathophysiology and accelerate the development of targeted therapies. Finally, the integration of artificial intelligence (AI) and machine learning in RA research is still in its infancy. Advanced computational tools can decode complex cell death-immune networks, predict TCM compound synergies, or stratify patients for personalized therapies. However, current efforts lack interdisciplinary collaboration, which limits the interpretability and clinical utility of AI-driven models.

In addition to mechanistic advances, several translational challenges must be addressed to move these discoveries toward clinical application. First, the development of exosomal ncRNAs as reliable biomarkers is currently hampered by technical barriers (including low enrichment efficiency and limited sensitivity), which necessitate further methodological innovation. Second, the multi-target nature and poor bioavailability of TCM-derived therapies call for advanced delivery platforms; recent progress in nanotechnology-driven delivery systems (such as nanoparticles and liposomes) holds promise for improving specificity and efficacy of both TCM components and ncRNA-based therapeutics. Third, synergistic approaches that combine cell death pathway inhibitors (e.g., Necrostatin-1) with ncRNA modulators may overcome therapeutic resistance and enhance treatment outcomes in RA. Addressing these clinical and technical barriers will be crucial for translating mechanistic insights into effective precision therapies for RA.

This work not only provides a comprehensive framework for understanding cell death mechanisms in RA, but also underscores the translational value of ncRNA-based diagnostics and TCM-inspired therapeutics for combating this complex autoimmune disorder.
